# Sex differences in neurocognitive response to metacognitive training in first-episode psychosis: Implications for personalized interventions

**DOI:** 10.1007/s00737-025-01637-3

**Published:** 2026-02-09

**Authors:** Marina Verdaguer-Rodríguez, Josep Oriol Comas, Raquel López-Carrilero, Luciana Díaz-Cutraro, Victoria Espinosa, Isabel Ruiz-Delgado, María Luisa Barrigón, Eva Grasa, Esther Pousa, Fermín González-Higueras, Jordi Cid, Esther Lorente-Rovira, Ana Barajas, Spanish Metacognition Study Group, A. Acevedo, A. Alonso-Solís, J. Anglès, L. Ansó, M. A. Argany, A. Aznar, M. L. Barrigón, M. Beltrán, I. Birulés, J. L. Bogas, N. Camprubí, M. Carbonero, E. Carrasco, R. Casañas, J. Cid, E. Conesa, I. Corripio, P. Cortes, A. de Apraiz, M. Delgado, L. Domínguez, M. J. Escartí, A. Escudero, I. Esteban Pinos, C. Franco, C. García, R. Gonzalez-Casares, F. González-Higueras, M. A. L. González-Montoro, E. González, E. Grasa-Bello, A. Guasp, M. E. Huerta-Ramos, P. Huertas, A. Jiménez-Díaz, L. L. Lalucat, B. Llacer, R. López-Carrilero, A. López-Frutos, E. Lorente, A. Luengo, N. Mantecón, L. Mas-Expósito, M. Montes, S. Moritz, E. Murgui, M. Nuñez, E. Palomer, T. Peláez, K. Planell, C. Planellas, P. Pleguezuelo-Garrote, E. Pousa, M. Renovell, N. Rodríguez, R. Rubio, I. Ruiz-Delgado, M. San Emeterio, E. Sánchez, S. Sánchez-Alonso, J. Sanjuán, B. Sans, H. Sió, M. Teixidó, P. Torres, M. Vidiella, M. A. Vila, R. Vila-Badia, F. Villegas, Susana Ochoa

**Affiliations:** 1 Etiopatogènia i Tractament dels Trastorns Mentals Greus (MERITT), Institut de Recerca Sant Joan de Déu, Esplugues de Llobregat, Spain; 2 Parc Sanitari Sant Joan de Déu, Sant Boi de Llobregat, Spain; 3 Department of Clinical and Health Psychology, Universitat Autònoma de Barcelona, Cerdanyola del Vallès, Spain; 4 Centro de Investigación Biomédica en Red de Salud Mental (CIBERSAM), ISCIII, Madrid, Spain; 5 Fundació Hospitalària de Mollet, Hospital de Mollet, Mollet del Vallès, Spain; 6 Unidad de Salud Mental Comunitaria Málaga Norte, UGC Salud Mental Carlos Haya, Servicio Andaluz de Salud, Málaga, Spain; 7 Institute of Psychiatry and Mental Health, Hospital General Universitario Gregorio Marañón, IiSGM, School of Medicine, Universidad Complutense., Madrid, Spain; 8 Mental Health, Institut de Recerca Sant Pau (IR SANT PAU), Barcelona, Spain; 9 Hospital de la Santa Creu i Sant Pau, Barcelona, Spain; 10 Hospital Universitario de Jaén, Servicio Andaluz de Salud, Jaén, Spain; 11 Mental Health & Addiction Research Group, IdiBGi, Institut d’Assistencia Sanitària, Girona, Spain; 12 Hospital Clínico Universitario de Valencia, , Valencia, Spain; 13 Serra Húnter Programme, Generalitat de Catalunya, Barcelona, Spain

**Keywords:** Psychosis, Cognition, Psychological Interventions, Gender, Intersectionality

## Abstract

**Purpose:**

Neurocognitive impairments are a core feature of psychosis and impact long-term outcomes. While sex differences in neurocognition have been observed in first-episode psychosis (FEP), findings remain mixed, and little is known about differential responses to metacognitive interventions. This study examined sex differences in the effectiveness of Metacognitive Training (MCT) on neurocognitive outcomes in FEP.

**Methods:**

A total of 122 individuals with FEP were randomized to receive either MCT or psychoeducational intervention. Neurocognitive performance was assessed at baseline and at 6-month follow-up using a comprehensive battery (CPT-II, TMT, WCST, Stroop test, TAVEC, WAIS-III Digit Span). General Linear Models tested the effects of intervention, sex, and their interaction, both unadjusted and adjusted for covariates.

**Results:**

MCT led to greater improvements than psychoeducation in immediate recall, processing speed, cognitive flexibility, inhibitory control, and attention. Improvements in immediate recall and Stroop Interference remained significant after adjustment, with Stroop performance also influenced by diagnosis. Across groups, men performed better in verbal memory, while women showed increased serial clustering in short-term recall, an effect that remained significant after adjustment. Notably, a group-by-sex interaction indicated that women receiving MCT experienced greater gains in short-term recall after controlling for covariates.

**Conclusions:**

MCT enhances specific neurocognitive functions in FEP and shows promising effects for women in verbal memory processes. These findings underscore the importance of incorporating sex and diagnostic factors when tailoring early interventions for psychosis and highlight the potential of MCT as a personalized cognitive strategy.

**Supplementary Information:**

The online version contains supplementary material available at https://doi.org/10.1007/s00737-025-01637-3.

## Introduction

Neurocognitive impairments are a core characteristic of psychosis, affecting domains such as attention, memory, executive functioning, and processing speed (Mesholam-Gately et al. 2009; Fatouros-Bergman et al. 2014; Tschentscher et al. 2023). These deficits can often emerge during first-episode psychosis (FEP) and tend to persist over time (Fatouros-Bergman et al. 2014; Albus et al. 2020; Tschentscher et al. 2023). Neurocognitive functioning also predicts transition from clinical high-risk states to psychosis (Catalan et al. 2021; Andreou et al. 2023), emphasizing its importance in prevention and early intervention.

An important consideration when investigating neurocognitive functioning in psychosis is sex differences. Although literature on this topic is growing, findings in FEP remain mixed. Some studies suggest men outperform women in visuospatial abilities (Ayesa-Arriola et al. 2014), verbal comprehension (Danaher et al. 2018), reasoning and problem solving (Labad et al. 2016), processing speed (Ayesa-Arriola et al. 2014; Serra-Navarro et al. 2022), and working memory (Ittig et al. 2015), whereas women tend to perform better in verbal memory (Ayesa-Arriola et al. 2014; Ittig et al. 2015; Pu et al. 2019; Buck et al. 2022; Palacios-Garran et al. 2025) and verbal fluency (Palacios-Garran et al. 2025). Conversely, other studies report no significant differences in verbal learning (Hui et al. 2016; Danaher et al. 2018), processing speed (Fatouros-Bergman et al. 2014; Pu et al. 2019; Labad et al. 2016) or working memory (Ayesa-Arriola et al. 2014; Danaher et al. 2018). Other studies report opposite patterns, such as better attention and processing speed in men (Serra-Navarro et al. 2022), while others favor women (Palacios-Garran et al. 2025).

Sex-effects also appear to influence the impact of neurocognition on other variables. In men with FEP, lower neurocognitive performance is associated with a stronger jumping to conclusions bias, a pattern not seen in women (Ferrer-Quintero et al. 2022; Díaz-Cutraro et al. 2025). For social cognition, both theory of mind and emotion recognition relate to cognitive flexibility and inhibitory control in men – while in women, theory of mind relates only to inhibition, and emotion recognition to multiple domains including working memory, selective attention, cognitive flexibility and inhibition (López-Carrilero et al. 2024). Poorer processing speed and executive dysfunction predict worse social functioning in women, but not in men (Serra-Navarro et al. 2022). However, evidence on sex differences in neuropsychological treatment response is limited. A longitudinal study suggested male sex predicted greater cognitive impairments ten years after early intervention for FEP (Bergh et al. 2016). Others demonstrated women with FEP showed greater neurocognitive improvements than men during early intervention, which particularly remained for negative symptoms in the long-term, but not for neurocognition (Ayesa-Arriola et al. 2020). These differences may be partially explained by distinct neurocognitive profiles. In men, verbal memory deficits predict negative symptoms; whereas in women, premorbid adjustment – but not neurocognition – appears more relevant (Mezquida et al. 2023).

Addressing neurocognitive deficits early during psychosis is critical. Metacognition has been proposed as a key mechanism linking neurocognition to functional outcomes (Kharawala et al, 2021; Davies et al. 2017), suggesting that targeting metacognition may enhance the effects of cognitive interventions, like Cognitive Remediation (CR) on social functioning skills (Davies et al. 2017; Reeder et al. 2017). In this regard, Metacognitive Training (MCT) is an evidence-based, structured group program combining psychoeducation, Cognitive Behavioral Therapy (CBT), and CR (Moritz et al. 2014). Originally developed to target cognitive biases associated with delusions (Moritz and Woodward 2007), MCT shows additional benefits for negative symptoms, self-esteem, and functioning (Penney et al. 2022; Meinhart et al. 2025; Goncalves et al. 2025). Moreover, emerging evidence suggests MCT may enhance neurocognition (Moritz et al. 2011; Wang et al. 2022; Fekete et al. 2022; Ruiz-Delgado et al. 2022; Goncalves et al. 2025). Yet, to date, no studies have explored whether neurocognitive response to MCT differs by sex.

A growing body of literature suggests that sex may influence treatment response in psychosis. Sex differences have been documented in clinical presentation, illness course, and functional outcomes, as well as in social cognition and neurocognitive profiles (Riecher-Rössler and Häfner 2000; Ochoa et al. 2012; Riecher-Rössler et al. 2018). Biological factors, such as the modulatory effects of estrogen on symptoms and cognitive functioning, have also been proposed as potential moderators of treatment response (Seeman 2006; Riecher-Rössler and Kulkarni 2011; Mu et al. 2024). Moreover, evidence indicates that neurocognition and social cognition influence functional outcomes after psychosocial interventions (Brekke et al. 2007). Therefore, if men and women differ in these cognitive domains, such differences may contribute to how they respond to treatments. Taken together, these distinctions may shape how individuals with psychosis engage with and benefit from psychosocial and cognitive interventions (Moniem and Kafetzopoulos 2025). In the context of MCT specifically, some evidence suggests that some of its components, particularly those involving cognitive insight, may operate differently across sexes. A study by Salas-Sender et al. (2020) found that women with FEP benefited more from MCT than their male counterparts, showing greater reductions in the personalizing bias, an effect not observed in the psychoeducational control group. Together with the mixed literature on sex differences in neurocognition in FEP and the increasing interest on personalized intervention strategies for early psychosis (Breitborde et al. 2017; Mazza et al. 2021), these findings support the rationale for examining whether sex-specific patterns also emerge in neurocognitive response to MCT.

Therefore, this study aims to explore sex differences in neurocognitive outcomes in FEP following MCT. We hypothesize that MCT will improve neurocognition across sexes, with potential sex-related differences in the pattern of improvements. Given the exploratory nature of this study and the mixed findings in the literature, we refrain from establishing a directional hypothesis regarding sex effects.

## Materials and methods

### Design

This study analyzed baseline and follow-up data of a previous multicentric single-blinded randomized clinical trial with two parallel intervention groups (Clinical Trials Identifier NCT02340559). Participants were randomized to either the experimental group (MCT) or the control group (psychoeducation group sessions), using a random number table. More details on the RCT design are described elsewhere (Ochoa et al. 2017).

### Participants

The sample comprised 122 FEP outpatients, within a maximum of five years since symptom onset (Breitborde et al. 2009). Participants were recruited from nine mental health centers in Spain (see Online Resource 1) and were informed of the study's characteristics. If they accepted, participants signed the informed consent form. Trained psychologists, blinded to the group condition of the participants, conducted the assessments at baseline, post-treatment and follow-up (6 months after finishing treatment), between 2015 and 2017. Concordance indexes in the assessment were satisfactory (Ochoa et al. 2017). Inclusion and exclusion criteria are outlined in Online Resource 2. The study followed the World Medical Association's Declaration of Helsinki, and approved by the ethics committee of Sant Joan de Déu (PIC-73–11) and the ethics committees of each participating center.

### Measures

Descriptive data of the sample were collected with an ad hoc sociodemographic questionnaire. Antipsychotic medication dosages were converted to chlorpromazine equivalents. Symptom severity was assessed with the Positive and Negative Syndrome Scale (PANSS) (Kay et al. 1987; Peralta and Zorita 1994) with the Emsley 7-factor solution (Emsley et al. 2003). Global Assessment of Functioning Scale (GAF) (Endicott 1976) was used to assess global and clinical functioning. Scale of Unawareness of Mental Disorders (SUMD) (Amador et al. 1993; Ruiz et al. 2008) was used to measure clinical insight.

Neurocognitive domains were measured with a battery of tests. The scores were standardized following the Spanish validation of the instruments, with a mean of 50 and a standard deviation of 10. The Continuous Performance Test for Windows (CPT-II) (Conners 2000) was used to assess attention. The Trail Making Test (TMT) (Reitan 1958; Fernández et al. 2002) was used to measure processing speed (TMT-A), cognitive flexibility and response inhibition (TMT-B). The computerized version of the Wisconsin Card Sorting Test (WCST) (Grant and Berg 1948; Tien et al. 1996) was used to assess executive functions, such as planning, cognitive flexibility, response inhibition, and perseveration. The Stroop Test (Stroop 1935; Golden 2001) was used to measure processing speed (word and color conditions), selective attention, cognitive flexibility, and response inhibition (color-word condition and interference score). The Spanish version of the California Verbal Learning Test (TAVEC) (Delis et al. 1988; Benedet and Alejandre 1998) was used to assess verbal learning and memory. The Digit Span subtest of the Wechsler Adult Intelligence Scale (WAIS-III) (Wechsler 1999; Gonzalez-Blanch et al. 2011) was used to measure working memory. Premorbid IQ was obtained with the Vocabulary subtest of the WAIS-III, to evaluate one of the exclusion criteria (see Online Resource 2). Online Resource 3 contains further details about items, score ranges and their interpretation, of clinical and neurocognitive tests.

### Interventions

The MCT was delivered in its 3rd edition using the module set A and consisted of 1-h weekly group sessions over8 weeks. Sessions followed a structured format supported by PowerPoint presentations, which were translated into Spanish and validated by researchers from the Spanish Metacognition Group. Each session addressed metacognitive aspects related to common cognitive biases in psychosis, including: Attributional Style (1 session), Jumping to Conclusions (2 sessions), Changing Beliefs (1 session), Theory of Mind and Emotional Recognition (2 sessions), Memory Errors (1 session), and Depression and Mood (1 session). Details on the modules content can be found in the Online Resource 4 (Moritz et al. 2010). The sessions were conducted by psychologists and psychiatrists who had been previously trained by Steffen Moritz’s and Lisa Schilling’s team from the Universitätsklinikum Hamburg-Eppendorf (UKE). The control group participated in 1-h weekly psychoeducation group sessions, for 8 weeks, covering healthy habits, risk behaviors, relapse prevention, video forums, employment and leisure resources, and community support. Both interventions were delivered during the same period of time and took place in the participant’s usual mental health centers, in groups of 4 to 8 participants.

### Statistical analyses

Analyses were conducted using SPSS Statistics v26. We tested data normality with Kolmogorov–Smirnov or Shapiro–Wilk test. Sex differences in sociodemographic and clinical variables were analyzed using Chi-square tests for categorical variables, and *t*-tests or Mann–Whitney *U* tests for continuous variables, when appropriate, with *p* < 0.05 as significance threshold. We obtained effect sizes with Cohen’s *d*.

We examined the effects of sex, intervention group, and their interaction, on neurocognitive performance over time using repeated-measures General Linear Models (GLM), comparing baseline and follow-up scores. For tasks with post-treatment data available (TMT-B, WCST, Digit Span), three time points were analyzed. GLMs were first run unadjusted, with group and sex as fixed factors, then adjusted for sociodemographic and clinical variables that showed significant sex differences at baseline. Diagnosis was recoded into two categories: Schizophrenia and Other Psychotic Disorders, the latter including schizoaffective disorder, schizophreniform disorder, delusional disorder, brief psychotic disorder, or psychotic disorder not otherwise specified. Effect sizes were obtained with partial eta squared (η_p_^2^), interpreted as small (0.01), moderate (0.06), or large (0.14) (Cohen et al. 2001). We did not perform multiple comparisons corrections due to the exploratory nature of the study (Bender and Lange 2001; Nakagawa 2004).

## Results

Table 1 shows sociodemographic and clinical characteristics of the sample by sex. A total of 122 participants were included: 85 men (69.67%) and 37 women (30.33%). 89 participants completed post-treatment assessments, and 81 remained for follow-up. The dropout rate was 27.5%, with similar rates across groups. Compared to men, women had a later age of symptom onset (*p* = 0.026) and better clinical insight scores (*p* = 0.035). Diagnostic distribution also differed by sex (*p* = 0.017). Schizophrenia was more common in men, while women were more frequently diagnosed with other psychotic spectrum disorders. Since these variables were significant, they were included as covariates in the adjusted GLM.

**Table 1:** Description of sociodemographic and clinical variables of the sample, regarding sex

	Men (*n* = 85)	Women (*n* = 37)	Statistic (U-MW/t/X^2^)	*p*-value	Cohen’s *d*	95% CI	
						LL	UL
	Mean (SD)	Mean (SD)					
Age (years)	26.64 (6.76)	29.78 (8.36)	1.958^a^	.050	-0.431	-6.26	-0.02
Age at symptom onset	24.27 (6.79)	27.57 (7.79)	2.226^a^	.026*	-0,464	-6.25	-0.35
Education level (%)			6.413^b^	.273			
Incomplete primary education	16.5%	8.1%					
Complete primary education	22.4%	21.6%					
Incomplete secondary education	21.2%	13.5%					
Complete secondary education	22.4%	21.6%					
Incomplete superior education	8.2%	10.8%					
Complete superior education	9.4%	24.3%					
Diagnosis (DSM-IV-TR) (%)			6.394^b^	.017*			
Schizophrenia	51.8%	27.0%					
Other psychotic disorders	48.2%	73.0%					
Antipsychotic medication^d^	537.56 (718.49)	398.21 (330.10)	-1.140^a^	.254	0,222	-48.68	327.3
PANSS Emsley factors							
Negative	15.49 (7.00)	13.32 (5.91)	-1.549^a^	.121	0,324	-0.28	4.62
Positive	14.63 (5.43)	14.30 (5.36)	0.313^c^	.755	0,061	-1.78	2.45
Disorganized	7.91 (2.85)	7.57 (3.26)	-1.278^a^	.201	0,114	-0.90	1.58
Excited	5.04 (1.56)	4.60 (0.99)	-1.158^a^	.247	0,311	-0.03	0.91
Motor	2.65 (1.17)	2.43 (0.77)	-0.401^a^	.689	0,206	-0.14	0.58
Depression	4.10 (2.04)	4.57 (2.29)	0.983^a^	.326	-0,222	-1.34	0.40
Anxiety	5.62 (1.92)	5.32 (2.02)	-0.800^a^	.424	0,154	-0.48	1.08
GAF	61.08 (12.95)	61.68 (12.56)	0.028^a^	.977	-0,047	-5.58	4.38
SUMD (global)	5.70 (3.41)	4.73 (3.52)	-2.111^a^	.035*	0,282	-0.40	2.34

*Abbreviations: CI* Confidence Interval, *DDD* Defined Daily Dose, *GAF* Global Assessment of Functioning, *LL* lower limit, *PANSS* Positive and Negative Syndrome Scale, *SUMD* Scale Unawareness of Mental Disorders, *UL* upper limit

^a^Mann–Whitney *U* test

^b^Chi-square test

^c^*T*-test

^*d*^Antipsychotic doses are expressed as chlorpromazine equivalence (mg/d)

^*^Level of significance < 0.05

Description of the neurocognitive outcomes at baseline and follow-up are displayed in Table 2. The first GLM examining the effectiveness of MCT on neurocognition by sex is presented in Online Resource 5, and the full GLM adjusted for covariates is shown in Table 3. In the unadjusted GLM, sex effects were observed in several verbal memory outcomes. Across groups, men improved more than women in short-term cued recall (*p* = 0.030; η_p_^2^ = 0.061) and made fewer intrusions in free recall (*p* = 0.025; η_p_^2^ = 0.065). Women, in turn, showed greater use of serial clustering in short-term free recall (*p* = 0.005; η_p_^2^ = 0.103). After adjusting for covariates, the sex effect in serial clustering remained significant (*p* = 0.001; η_p_^2^ = 0.173), also explained by a significant diagnosis effect (*p* = 0.028; η_p_^2^ = 0.081). We found a significant group effect in the first presentation of the immediate recall task (*p* = 0.039; η_p_^2^ = 0.057), with MCT yielding greater improvement for both sexes. This effect remained significant after covariate adjustment (*p* = 0.003; η_p_^2^ = 0.144). Also, when controlling for covariates, a significant sex-by-group interaction emerged in short-term free recall (*p* = 0.043; η_p_^2^ = 0.068), with women in the MCT group showing greater improvement.

**Table 2:** Description of neurocognitive variables at baseline and follow-up, after controlling for covariates, regarding sex

	Psychoeducational group				MCT group			
	Baseline *Mean (SD)*		Follow-up *Mean (SD)*		Baseline *Mean (SD)*		Follow-up *Mean (SD)*	
	Men	Women	Men	Women	Men	Women	Men	Women
**CPT**^**b**^								
Omissions	98.49 (132.02)	57.14 (16.02)	81.66 (75.28)	68.66 (42.23)	87.03 (53.59)	121.66 (145.84)	62.52 (28.76)	150.36 (315.08)
Commissions	50.80 (11.88)	57.02 (11.25)	49.64 (10.69)	59.77 (10.26)	53.48 (12.16)	55.81 (11.58)	52.07 (13.87)	51.42 (14.97)
Hit Index Reaction Time	59.55 (14.40)	52.54 (9.86)	61.57 (18.51)	55.81 (13.91)	56.10 (14.93)	59.62 (21.6)	53.08 (14.47)	57.26 (22.64)
**TMT**^**a**^								
TMT-A	70.34 (19.77)	59.10 (11.46)	60.42 (17.79)	53.59 (12.87)	59.61 (11.49)	62.83 (14.86)	55.05 (11.53)	57.54 (15.14)
TMT-B	75.98 (22.82)	57.90 (11.64)	68.04 (26.74)	53.01 (16.61)	68.70 (20.64)	65.40 (16.48)	57.75 (11.02)	62.46 (22.30)
**WCST**^**a**^								
Perseverative errors	47.04 (14.22)	44.56 (6.67)	50.26 (6.92)	47.44 (6.48)	44.00 (7.37)	45.73 (8.62)	48.75 (6.13)	45.73 (9.86)
Non-perseverative errors	46.09 (14.42)	42.56 (9.19)	46.78 (12.00)	44.89 (9.10)	40.69 (8.40)	45.73 (5.55)	48.88 (7.54)	47.36 (9.83)
Total errors	45.43 (14.93)	42.88 (6.85)	47.57 (10.52)	45.75 (7.27)	41.56 (8.28)	45.82 (6.37)	49.19 (9.15)	46.73 (13.84)
**Stroop Test**^**a**^								
Word	41.70 (9.83)	44.67 (7.28)	41.65 (10.02)	44.33 (10.40)	41.05 (11.92)	45.33 (10.85)	44.60 (11.50)	48.22 (10.70)
Color	34.70 (9.14)	36.67 (8.21)	37.09 (9.23)	37.92 (8.67)	39.40 (9.94)	40.00 (8.00)	40.60 (10.19)	46.00 (16.58)
Word-Color	47.43 (13.70)	43.42 (10.31)	42.74 (9.81)	44.67 (11.80)	49.70 (14.78)	48.78 (16.75)	46.05 (12.69)	43.00 (9.41)
Interference	55.87 (10.78)	53.83 (6.94)	54.65 (8.75)	54.92 (10.02)	58.25 (11.10)	56.56 (15.90)	53.70 (8.42)	48.11 (8.98)
**WAIS-III Digit Span Subtest**^**a**^	43.74 (12.02)	44.30 (9.42)	47.03 (11.92)	46.27 (11.96)	43.47 (8.94)	49.18 (9.01)	42.16 (9.63)	48.73 (12.23)
**CVLT**^**a**^								
Immediate recall								
*First presentation (A1)*	39.12 (9.97)	47.38 (13.84)	41.32 (9.45)	45.62 (17.08)	37.79 (7.99)	39.64 (6.93)	45.36 (9.43)	47.18 (8.64)
*Fifth presentation (A5)*	33.58 (18.05)	43.50 (17.15)	38.82 (19.07)	46.10 (13.04)	33.28 (13.62)	39.41 (14.81)	39.35 (13.38)	42.47 (13.71)
*Total (A1-A5)*^*b*^	33.55 (13.95)	45.14 (13.24)	39.26 (15.04)	44.32 (14.16)	34.52 (10.21)	37.04 (8.42)	40.10 (11.52)	43.88 (14.07)
Free recall								
*Short-term*	35.30 (14.09)	46.15 (11.98)	41.45 (14.31)	45.87 (13.96)	36.05 (12.99)	38.26 (11.30)	37.99 (12.70)	44.84 (15.73)
*Long-term*	33.09 (16.25)	42.39 (12.37)	38.59 (19.52)	44.79 (15.38)	33.80 (12.39)	39.16 (12.20)	37.04 (12.63)	44.29 (15.70)
Cued recall								
*Short-term*	34.94 (14.33)	41.24 (14.30)	41.73 (17.47)	41.62 (17.60)	34.62 (12.71)	38.31 (14.00)	37.82 (13.12)	40.63 (16.30)
*Long-term*	33.04 (19.08)	40.32 (16.30)	41.10 (18.00)	43.52 (17.66)	31.42 (13.90)	35.98 (13.31)	38.10 (13.82)	39.54 (17.33)
Semantic clustering								
*Immediate recall*	40.17 (7.33)	48.45 (10.35)	48.92 (31.14)	46.47 (10.36)	41.48 (4.22)	40.61 (5.90)	42.35 (4.84)	45.74 (13.35)
*Short-term free recall*	39.75 (8.24)	48.74 (8.91)	43.43 (10.49)	46.40 (11.13)	41.76 (7.25)	43.44 (8.00)	43.05 (8.31)	44.21 (9.60)
*Long-term free recall*	38.75 (8.28)	45.12 (8.39)	44.41 (12.75)	46.61 (10.21)	39.98 (7.76)	42.81 (8.24)	40.83 (8.29)	45.54 (10.84)
Serial clustering								
*Immediate recall*	49.42 (7.75)	50.59 (6.70)	49.81 (10.33)	49.58 (4.41)	55.19 (19.13)	53.74 (6.83)	53.15 (9.71)	53.14 (14.78)
*Short-term free recall*	51.44 (6.35)	46.88 (3.72)	49.65 (6.81)	51.28 (11.48)	55.65 (16.00)	47.33 (3.94)	51.00 (7.32)	54.33 (12.35)
*Long-term free recall*	47.33 (4.78)	52.27 (6.13)	48.74 (6.17)	52.18 (8.14)	51.93 (14.36)	50.75 (9.12)	54.48 (11.96)	52.78 (15.76)
Perseverations	48.01 (11.00)	44.45 (6.90)	48.45 (11.34)	44.74 (6.75)	53.38 (9.16)	52.74 (10.03)	49.45 (9.06)	52.19 (12.85)
Intrusions								
*Free recall*	50.28 (9.56)	46.51 (6.31)	47.73 (8.73)	51.70 (11.83)	54.14 (13.08)	48.20 (7.69)	48.30 (8.55)	47.33 (5.69)
*Cued recall*	52.72 (10.94)	52.44 (14.08)	47.20 (5.33)	49.88 (7.87)	52.59 (11.59)	51.71 (14.90)	48.53 (5.54)	47.97 (6.75)
Recognition								
*Accuracy*	32.29 (27.18)	45.50 (17.94)	35.44 (30.40)	45.82 (12.57)	38.67 (16.72)	43.17 (15.89)	42.46 (10.06)	45.73 (17.80)
*False positives*	59.97 (19.04)	49.68 (8.94)	57.52 (26.65)	49.66 (6.74)	53.38 (11.45)	50.66 (7.15)	53.27 (9.67)	51.67 (14.97)

*Abbreviations: CPT* Continuous Performance Test, *CVLT* California Verbal Learning Test, *MCT* Metacognitive Training, *SD* Standard Deviation, *TMT* Trail Making test, WAIS-III, Wechsler Adult Intelligence Scale, *WCST* Wisconsin Card Sorting Test

^*a*^Values are shown as T scores (mean = 50, SD = 10)

^*b*^Total = sum of trials A1 to A5

Group effects were also found in the Word (*p* = 0.029; η_p_^2^ = 0.064) and Interference (*p* = 0.031; η_p_^2^ = 0.062) conditions of the Stroop Test in the initial GLM, favoring MCT. After covariate adjustment, the Interference condition remained significant (*p* = 0.040; η_p_^2^ = 0.072), along with a significant diagnosis effect (*p* = 0.034; η_p_^2^ = 0.076). An initial group effect was observed for the CPT Hit Index Reaction Time (*p* = 0.044; η_p_^2^ = 0.057), with greater improvement in the MCT group, though this effect did not remain after covariate adjustment. No other neurocognitive variables showed significant effects of sex, group, or interaction (Table 3).

**Table 3:** Effects of sex and intervention group on neurocognitive variables over time, adjusted for covariates

	**Comparison between groups (baseline vs follow-up)**					
	*P-value (effect size)*					
	Group effect	Sex effect	Group*Sex	Age at onset	Diagnosis	Clinical insight
**CPT**						
Omissions	.901 (.000)	.128 (.042)	.774 (.002)	.275 (.022)	.388 (.014)	.414 (.012)
Commissions	.189 (.031)	.635 (.004)	.122 (.043)	.527 (.007)	.126 (.042)	.995 (.000)
Hit Index Reaction Time	.133 (.041)	.643 (.004)	.993 (.000)	.392 (.013)	.896 (.000)	.497 (.008)
**TMT**						
TMT-A	.334 (.016)	.853 (.001)	.248 (.023)	.044* (.067)	.584 (.005)	.941 (.000)
TMT-B	.905 (.000)	.278 (.021)	.705 (.003)	.363 (.015)	.665 (.003)	.882 (.000)
**WCST**						
Perseverative errors	.806 (.001)	.381 (.015)	.487 (.009)	.680 (.003)	.926 (.000)	.964 (.000)
Non-perseverative errors	.355 (.016)	.292 (.021)	.180 (.034)	.127 (.004)	.936 (.000)	.693 (.003)
Total errors	.649 (.004)	.209 (.031)	.294 (.022)	.280 (.023)	.586 (.006)	.998 (.000)
**Stroop Test**						
Word	.139 (.038)	.602 (.005)	.875 (.000)	.785 (.001)	.535 (.007)	.233 (.025)
Color	.415 (.012)	.611 (.005)	.166 (.033)	.533 (.007)	.666 (.003)	.927 (.000)
Word-Color	.389 (.013)	.147 (.036)	.067 (.058)	.606 (.005)	.006* (.124)	.863 (.001)
Interference	.040* (.072)	.601 (.005)	.133 (.039)	.582 (.005)	.034* (.076)	.677 (.003)
**WAIS-III Digit Span Subtest**	.108 (.045)	.769 (.002)	.431 (.011)	.621 (.004)	.158 (.035)	.276 (.021)
**CVLT**						
Immediate recall						
*First presentation*	.003* (.144)	.536 (.007)	.452 (.010)	.648 (.004)	.777 (.001)	.111 (.043)
*Fifth presentation*	.698 (.003)	.750 (.002)	.625 (.004)	.526 (.007)	.213 (.026)	.048* (.065)
Total (A1-A5)**	.098 (.046)	.396 (.012)	.222 (.021)	.545 (.006)	.365 (.014)	.169 (.032)
Free recall						
*Short-term*	.560 (.006)	.986 (.000)	.043* (.068)	.862 (.001)	.302 (.018)	.631 (.004)
*Long-term*	.610 (.004)	.597 (.005)	.853 (.001)	.205 (.027)	.046* (.066)	.886 (.000)
Cued recall						
*Short-term*	.748 (.002)	.173 (.031)	.310 (.017)	.847 (.001)	.961 (.000)	.882 (.000)
*Long-term*	.871 (.000)	.200 (.028)	.965 (.000)	.439 (.010)	.207 (.027)	.731 (.002)
Semantic clustering						
*Immediate recall*	.932 (.000)	.504 (.008)	.251 (.022)	.262 (.021)	.383 (.013)	.706 (.002)
*Short-term free recall*	.873 (.000)	.231 (.024)	.227 (.025)	.613 (.004)	.945 (.000)	.886 (.000)
*Long-term free recall*	.400 (.012)	.299 (.018)	.205 (.027)	.120 (.041)	.697 (.003)	.977 (.000)
Serial clustering						
*Immediate recall*	.865 (.000)	.453 (.010)	.993 (.000)	.417 (.011)	.136 (.037)	.393 (.012)
*Short-term free recall*	.977 (.000)	.001* (.173)	.738 (.002)	.757 (.002)	.028* (.081)	.908 (.000)
*Long-term free recall*	.499 (.008)	.778 (.001)	.756 (.002)	.947 (.000)	.062 (.058)	.625 (.004)
Perseverations	.242 (.023)	.708 (.002)	.352 (.015)	.927 (.000)	.407 (.012)	.956 (.000)
Intrusions						
*Free recall*	.060 (.059)	.072 (.054)	.766 (.002)	.499 (.008)	.626 (.004)	.086 (.049)
*Cued recall*	.879 (.000)	.456 (.009)	.860 (.001)	.140 (.037)	.012* (.102)	.046* (.066)
Recognition						
*Accuracy*	.589 (.005)	.726 (.002)	.580 (.005)	.962 (.000)	.004* (.132)	.960 (.000)
*False positives*	.692 (.003)	.893 (.000)	.920 (.003)	.875 (.000)	.095 (.047)	.027* (.081)

*Abbreviations: CPT* Continuous Performance Test, *CVLT* California Verbal Learning Test, *MCT* Metacognitive Training, *SD* Standard Deviation, *TMT* Trail Making test, *WAIS-III* Wechsler Adult Intelligence Scale, *WCST* Wisconsin Card Sorting Test

^*^Level of significance < 0.05

^**^ Total = sum of trials A1 to A5

Effect sizes are provided with partial eta square (η_p_^2^)

## Discussion

The present study provides novel insights into sex differences in neurocognitive response to MCT in individuals with FEP. Our results reveal both sex-specific and sex-independent neurocognitive improvements following MCT, suggesting implications for the personalization of early interventions in FEP. In line with our hypotheses, both men and women benefited from MCT in terms of neurocognition, with some improvements differing by sex, particularly in verbal memory and learning. Women in the MCT group presented greater enhancement in short-term free recall, aligning with previous research showing women with FEP tend to outperform men in verbal memory (Ayesa-Arriola et al. 2014; Ittig et al. 2015; Pu et al. 2019; Buck et al. 2020; Smelror et al. 2021; Palacios-Garran et al. 2025). Other sex-related effects in verbal memory were not specific to MCT and also appeared in the psychoeducation group, such as men improving more in short-term cued recall and making fewer intrusions during free recall. Given that women with FEP often outperform men in verbal memory, one might assume that men, having greater room for improvement, could benefit similarly from MCT or psychoeducation without requiring a specialized neurocognitive approach. Conversely, women may need interventions more directly targeting verbal memory to achieve significant gains, such as CR which is suggested to be particularly effective for improving verbal memory in psychosis (Miley et al. 2020; Vita et al. 2021). Whether combining MCT and CR enhances outcomes, especially in women, remains an open question. Although this hypothesis should be interpreted cautiously, it supports the importance of considering sex effects when designing early psychosis treatments. Future research should explore moderating variables and account for practice effects (Goldberg et al., 2010) that might explain improvements seen across groups.

The effect of sex on serial clustering in short-term free recall in women persisted after covariate adjustment, underscoring the robustness of this finding. Serial clustering involves recalling words in the original order of presentation and, although it is considered a less efficient strategy due to its reliance on working memory resources (Brébion et al. 1997, 2000), an increased use may also reflect enhanced engagement with task structure and strategic organization. Notably, this increase did not occur at the expense of semantic clustering, which also improved, though not significantly. This suggests greater cognitive flexibility, as women may be integrating multiple encoding strategies after treatment. Semantic clustering – recalling words by category – is associated with more effective verbal memory (Brébion et al. 2004) and better cognitive outcomes in psychosis (Vaskinn et al. 2007; Polyn et al. 2009; Gsottschneider et al. 2011; Gill et al. 2018; Brunet et al. 2020). Thus, increased serial clustering alongside a maintained use of semantic clustering may indicate a positive treatment response in women with FEP. Further investigation into sex-related differences in encoding strategies could clarify mechanisms behind verbal memory performance and the potential role in treatment response in FEP.

As expected, MCT was associated with greater gains than psychoeducation in multiple neurocognitive domains for both sexes. This supports prior research highlighting MCT’s broader cognitive benefits beyond reducing metacognitive biases (Moritz et al. 2011; Wang et al. 2022; Ruiz-Delgado et al. 2022). Specifically, we found a significant improvement in immediate recall following MCT, which remained after covariate adjustment. This is consistent with findings that MCT enhances verbal memory in schizophrenia (Wang et al. 2022; Fekete et al. 2022) and FEP (Ussorio et al. 2016; Ruiz-Delgado et al. 2022). Given verbal memory seems to predict functional outcomes and has proven to distinguish between treatment-responsive and treatment-resistant patients (Lepage et al. 2014; Fu et al. 2017; Millgate et al. 2022), early treatments should address deficits in this neurocognitive domain. Additionally, a significant group effect remained after adjustment in the Stroop Interference condition, suggesting MCT may also enhance cognitive flexibility and inhibitory control. Improving executive function is important, as it is strongly linked to metacognition (García-Mieres et al. 2020) and is often impaired in FEP (Riley et al. 2000; González et al. 2018). Our results support prior similar findings in FEP (Ussorio et al. 2016; Ruiz-Delgado et al. 2022), positioning MCT as a feasible intervention that enhances neurocognition through metacognitive abilities. Improvements after MCT in attention and processing speed were also observed in CPT Hit Index Reaction Time and Stroop Word condition scores, respectively. While other studies have reported similar effects (Moritz et al. 2011; Ussorio et al. 2016; Wang et al. 2022; Ruiz-Delgado et al. 2022), our adjusted analyses suggest influences beyond group effects alone, highlighting the need to further investigate moderators of MCT’s impact on attention and processing speed.

Beyond the specific links with MCT, the literature on neurocognitive outcomes after treatment provides a broader context for interpreting these findings. Verbal memory consistently emerges as one of the most treatment-responsive cognitive domains in psychosis. It can distinguish between treatment-responsive and treatment-resistant patients (Millgate et al. 2022). Impaired immediate verbal recall predicts non-remission and long-term functional disability in individuals at clinical high risk (CHR) (Hedges et al. 2022). Verbal memory performance also predicts transition to psychosis (Andreou et al. 2023). Similarly, cognitive remediation (CR) studies report robust gains in verbal learning and memory (Fiszdon et al. 2016; Miley et al. 2020). Improvements in this domain appear to contribute most to functional enhancement in early psychosis (Eack et al. 2011). In contrast, inhibitory control and related executive functions tend to be more resistant to intervention. Patients with treatment-resistant early psychosis show more severe deficits in cognitive control than treatment responders (Thomas et al. 2021), and difficulties inhibiting alternative responses have been linked to greater likelihood that patients continue to require intensive psychiatric care (Wykes and Van Der Gaag 2001). Evidence regarding cognitive flexibility as a predictor of outcomes is mixed. Some studies report a positive association with treatment outcomes (Garety et al. 1997), while others find no effect (Lincoln et al. 2014). Taken together, these findings underscore the relative responsiveness of verbal memory and the challenges in modifying inhibitory control. Within this broader context, the neurocognitive improvements observed after MCT in the present article may reflect enhancements in processes that are clinically meaningful and difficult to change.

Another key finding was the significant effect of diagnosis as a covariate in verbal memory, cognitive flexibility, and inhibitory control. The increase of serial clustering observed only in women may relate to the higher prevalence of schizophrenia among men. Schizophrenia, compared to other psychotic disorders, is associated with greater cognitive impairments and poorer verbal memory (Bora et al. 2009; Ayesa-Arriola et al. 2016; Buck et al. 2020). Thus, men in our sample may have experienced more difficulties integrating a broader variety of clustering strategies that would help them adapt to different tasks and contexts. This supports the idea that clinical heterogeneity in FEP interacts with sex to influence treatment response, consistent with evidence that neurocognitive predictors vary by sex and diagnosis (Mezquida et al. 2023).

Diagnosis may also moderate the impact of MCT on executive functioning, as both symptom severity and cognitive profiles can influence treatment response. In the present study, participants receiving MCT demonstrated greater gains in executive functions, particularly cognitive flexibility and inhibitory control, with diagnosis emerging as a significant covariate. This finding is consistent with evidence that neurocognitive impairments and cognitive biases interact to shape treatment response in psychosis. For instance, individuals with a stronger jumping to conclusions (JTC) bias appear to benefit more from MCT in terms of symptom reduction (Leanza et al. 2020), raising the question of whether different levels of JTC similarly moderate improvements in executive functioning. Previous research has linked JTC and cognitive control, which encompasses cognitive processes such as flexibility and response inhibition (Woodward et al. 2009; Shih et al. 2025). Moreover, individuals with schizophrenia generally show more severe deficits in cognitive flexibility compared to those with other psychosis-spectrum diagnoses (Zabala et al. 2008). Taken together, the observed diagnostic effect suggests that individuals with schizophrenia, who tend to show greater baseline executive dysfunction, may have experienced more benefits from MCT in inhibitory control and cognitive flexibility.

However, the role of diagnosis in MCT’s neurocognitive response remains understudied. Existing FEP studies vary in diagnostic inclusion criteria – some focus only on schizophrenia (Zhang et al. 2012; Ittig et al. 2015; Pu et al. 2019), others include affective psychoses (Danaher et al. 2018; Albus et al. 2020; Buck et al. 2020), or omit diagnostic details entirely. Future research should explore whether MCT is broadly effective or more suitable for specific diagnoses in FEP. Also, analyses could be stratified by both diagnosis and sex to examine subgroup-specific treatment outcomes. Given the relevance of diagnostic effects, adopting an intersectional approach (Crenshaw 1989; Subramaniapillai et al. 2024) that considers both sociodemographic and clinical factors would help capture how they interact to influence cognitive performance. Such an approach would move beyond examining these variables in isolation, better understanding of their effects on treatment response and supporting more personalized treatments in FEP by addressing specific needs based on sex and diagnosis.

Additionally, clinical insight emerged as a significant covariate influencing verbal memory performance. This finding aligns with evidence linking insight, memory, and metacognition in psychosis. Prior research shows that poorer insight is associated with deficits in verbal memory (Kitis et al. 2007; Tumkaya et al. 2009; Davies et al. 2017) and that metacognitive capacities may mediate this relationship (Lysaker et al. 2005; Kashyap et al. 2012). Individuals with better insight may engage more effectively with metacognitive interventions, allowing them to integrate new information and apply therapeutic strategies more efficiently. Our findings therefore suggest that verbal memory improvements following MCT may depend partly on individual differences in insight. Exploring the role of clinical insight in treatment response may help clarify the mechanisms underlying neurocognitive gains and support the development of more personalized intervention strategies. Some limitations to this study should be acknowledged. First, the sample was not balanced by sex. Due to convenience sampling based on availability and willingness to participate, sex distribution was not predetermined. Instead, it reflected natural recruitment resulting in a higher proportion of men, which aligns with the higher incidence of psychosis in men (McGrath et al. 2008). However, the smaller number of women may have limited power to detect significant improvements. Second, although our cognitive battery was broad, it did not assess domains such as visual memory or verbal fluency, which have been linked to MCT effectiveness (Moritz et al. 2011; Wang et al. 2022; Fekete et al. 2022; Ruiz-Delgado et al. 2022). Future studies on sex differences in treatment response should include those domains to have more comprehensive neurocognitive assessments. Third, our 6-month follow-up may have been too short to detect long-term neurocognitive effects of MCT. Prior studies have reported sustained effects of MCT on symptoms, cognitive biases, self-esteem, and functioning up to 1-year post-treatment (Moritz et al. 2014; Penney et al. 2022). Studies exploring MCT’s long-term impact on neurocognition are encouraged (Jeffrey et al. 2025). Lastly, hormonal fluctuations during the menstrual cycle may affect women’s cognitive performance (Hoff et al. 2001; Farage et al. 2008; Riecher-Rössler et al. 2018), but we lacked data on menstrual phase or hormonal treatment. Future studies should address this gap to clarify hormonal influences on female cognitive performance.

Despite these limitations, this is the first study to explore the effects of sex on neurocognitive response to MCT using an intersectional approach. Therefore, the present study has important implications for early treatment of psychosis. While neurocognition is not MCT’s primary treatment target, it shows promise as an intervention for FEP that enhances key neurocognitive domains, with both overall and sex-specific benefits. However, existing literature has not fully addressed sex differences, limiting generalizability (Jeffrey et al. 2025). From a clinical perspective, our results highlight the importance of incorporating sex and diagnosis into treatment planning. Given the central role of some neurocognitive domains in functional recovery, such as verbal memory and cognitive control, tailoring interventions regarding specific needs could optimize outcomes. For instance, integrating MCT with CR for FEP individuals could be an interesting way to possibility to maximize neurocognitive improvement. At the same time, improvements observed across groups point to the need for careful consideration of practice effects and additional moderators, including clinical insight, which may shape treatment response. Future research should further explore sex effects, adopting an intersectional perspective to examine how sociodemographic and clinical variables, such as diagnosis, influence cognitive improvements in FEP. It is also important to identify cognitive and metacognitive profiles that are most responsive to MCT, and to test combined interventions to clarify whether specific subgroups, particularly women or those with more severe forms of psychosis-spectrum diagnoses, derive differential benefit. Longitudinal studies will also be essential in determining how changes in neurocognition translate into functional gains, as well as advancing the personalization of early psychosis care. Providing evidence-based, cost-effective interventions adapted to patient profiles is crucial for optimizing treatment outcomes. Therefore, continued research in this direction is strongly encouraged.

## Supplementary Information

Below is the link to the electronic supplementary material.

Supplementary file1 (DOCX 45 KB)

## Data Availability

The data that support the findings of this study are not openly available due to reasons of confidentiality and are only available from the corresponding author upon reasonable request (S.O.; susana.ochoa@sjd.es).
