# Supplementary material for: Sex differences in neurocognitive response to metacognitive training in first-episode psychosis: Implications for personalized interventions
Source: Arch Womens Ment Health. 2026 Feb 9;29(1):36. doi: 10.1007/s00737-025-01637-3 (PMC12886316; doi:10.1007/s00737-025-01637-3)
Supplement: Supplementary file 1 — Supplementary file1 (DOCX 45 KB) [file 737_2025_1637_MOESM1_ESM.docx]

**Sex Differences in Neurocognitive Response to Metacognitive Training in First-Episode Psychosis: Implications for Personalized Interventions**

Marina Verdaguer-Rodríguez^1,2,3,4^; Comas, Josep Oriol Comas^1,2^; Raquel López Carrilero^1,2^; Luciana Díaz-Cutraro^1,2^; Victoria Espinosa^1,2^; Isabel Ruiz-Delgado^5^; María Luisa Barrigón^6^; Eva Grasa^4,7,8^; Esther Pousa^4,7^; Fermín González-Higueras^9^; Jordi Cid^10^; Esther Lorente-Rovira^4,11^; Ana Barajas^3,12,^*; Spanish Metacognition Study Group; Susana Ochoa^1,2,4,^*

**Share last authorship*

^1^ Etiopatogènia i Tractament dels Trastorns Mentals Greus (MERITT), Institut de Recerca Sant Joan de Déu, Esplugues de Llobregat, Spain

^2^ Parc Sanitari Sant Joan de Déu, Sant Boi de Llobregat, Spain

^3^ Department of Clinical and Health Psychology, Universitat Autònoma de Barcelona, Cerdanyola del Vallès, Spain

^4^ Centro de Investigación Biomédica en Red de Salud Mental (CIBERSAM), ISCIII, Madrid, Spain

^5^ Unidad de Salud Mental Comunitaria Málaga Norte, UGC Salud Mental Carlos Haya, Servicio Andaluz de Salud, Málaga, Spain

^6^ Institute of Psychiatry and Mental Health, Hospital General Universitario Gregorio Marañón, IiSGM, CIBERSAM, ISCIII, School of Medicine, Universidad Complutense, Madrid, Spain.

^7^ Mental Health, Institut de Recerca Sant Pau (IR SANT PAU), Sant Quintí 77-79, 08041 Barcelona, Spain

^8^ Hospital de la Santa Creu i Sant Pau, Sant Antoni Maria Claret 167, 08025 Barcelona, Spain

^9^ Hospital Universitario de Jaén, Servicio Andaluz de Salud, Jaén, Spain

^10^ Mental Health & Addiction Research Group, IdiBGi—Institut d’Assistencia Sanitària, Girona, Spain

^11^ Department of Child and Adolescent Psychiatry, Institute of Psychiatry and Mental Health, Hospital General Universitario Gregorio Marañón, IiSGM, School of Medicine, Universidad Complutense, Madrid, Spain

^12^ Serra Húnter Programme, Generalitat de Catalunya, Barcelona, Spain.

**Corresponding author: Susana Ochoa Güerre** [susana.ochoa@sjd.es](mailto:susana.ochoa@sjd.es)

C/ Dr. Pujades, 42, Sant Boi de Llobregat (08830), Barcelona, Spain

+34936406350 (extension 12538)

**Supplementary material**

**Online Resource 1: Participating Centers**

The present study was conducted as part of a multicenter clinical trial carried out across several mental health institutions in Spain. Below is a list of the participating centers that contributed to the recruitment and data collection, including the coordinating center:

- Servicio Andaluz de Salud de Jaen, Granada and Málaga
- Corporació Sanitària Parc Taulí
- Hospital de Santa Creu i Sant Pau
- Centre d’Higiene Mental Les Corts
- Institut d’Assistència Sanitària de Girona
- Hospital Clínico Universitario de Valencia
- Parc Sanitari Sant Joan de Déu (coordinating center)

**Online Resource 2: Inclusion and Exclusion Criteria**

Inclusion criteria were: (1) diagnosis of schizophrenia or other psychotic spectrum disorders according to DSM-IV-TR, such as schizoaffective disorder, schizophreniform disorder, delusional disorder, brief psychotic disorder, or psychotic disorder not otherwise specified; (2) <5 years from the onset of symptoms; (3) PANSS score of ≥3 in Delusions, Grandiosity, or Suspiciousness items, during the previous year; (4) age between 17 and 45. Exclusion criteria were: (1) traumatic brain injury, dementia, or intellectual disability (premorbid IQ ≤70, assessed with the Vocabulary subtest of the WAIS-III); (2) substance dependence (according to DSM-IV-TR); (3) PANSS score of ≥5 in Hostility and Uncooperativeness items, and of ≥6 in Suspiciousness item.

**Online Resource 3: Details on Items, Score Ranges and Interpretation of Clinical and Neurocognitive Tests**

- *PANSS* (Kay et al. 1987; Peralta and Zorita 1994): Higher scores indicate greater symptom severity. With the 7-factor solution (Emsley et al. 2003), we obtained the positive, negative, disorganized, excited, motor, depression, and anxiety factors. Factor scores range between the following:
  - Negative: 7 items, scores ranging from 7 to 49
  - Positive: 7 items, scores ranging from 7 to 49
  - Disorganized: 5 items, scores ranging from 5 to 37
  - Excited: 4 items, scores ranging from 4 to 28
  - Motor: 2 items, scores ranging from 2 to 14
  - Depression: 2 items, scores ranging from 2 to 14
  - Anxiety: 3 items, scores ranging from 3 to 21
- *GAF* (Endicott 1976): Higher scores indicate better functioning, ranging from 1 to 100.
- *SUMD* (Amador et al. 1993; Ruiz et al. 2008)*:* Higher scores indicate poorer clinical insight, ranging from 3 to 15.
- *CPT-II* (Conners 2000): We assessed Omissions, Commissions and Hit Index Reaction Time scores. Higher scores indicate a worse performance.
- *TMT* (Reitan 1958; Fernández et al. 2002)*:* Higher scores indicate a worse performance.
- *WCST* (Grant and Berg 1948; Tien et al. 1996): We included Perseverative, Non-Perseverative and Total Error scores. Higher scores indicate poorer performance.
- *Stroop Test* (Golden et al., 2001; Stroop et al., 1935): Higher scores indicate better performance.
- *TAVEC* (Benedet et al., 1998; Delis et al., 1988): We included immediate, free and cued recall, recognition, perseverations, intrusions, false positives, and encoding strategies. For immediate, free and cued recall, recognition, semantic clustering and serial clustering, higher scores indicate better performance. For perseverations, intrusions and false positives, higher scores indicate worse performance.
- *Digit Span subtest WAIS-III* (Wechsler et al., 1999; González-Blanch et al., 2011): Higher scores indicate better performance.

**Online Resource 4: Summary of MCT’s modules (3^rd^ edition)**

Adapted from Moritz and Woodward (2007), and the Metacognition Training Manual Program, Spanish Version (Moritz et al., 2010).

| Module | Target domain | Aim |
| --- | --- | --- |
| Module 1  Attribution: blaming and taking credit | Self-serving bias, depressive attributional style and monocausal attribution. | To consider multiple explanations for different situations by evaluating three possible sources: oneself, others, and situational factors (alone or in combination). Rather than guiding participants toward a single correct answer, the goal is to broaden their perspective and modify rigid attributional styles (e.g., consistently blaming oneself or always blaming others). The module also outlines the drawbacks of both depressive attributional patterns and self-serving biases, which can lower self-esteem or create interpersonal conflict. Overall, its core aim is to show that several factors may contribute to an event or situation, even when one explanation initially seems most obvious. |
| Module 2  Jumping to conclusions (I) | JTC, BADE | To avoid relying on first impressions, as they can lead to misinterpretations, errors, or partial truths. The module shows how situations evolve and that additional evidence may offer new insights. Therefore, alternative viewpoints should not be dismissed prematurely. Participants are encouraged to find a balanced approach between avoiding overly hasty decisions -which increase the risk of mistakes-, and excessive caution -which can cause unnecessary delays-. |
| Module 3  Changing beliefs | BADE | To counter the common tendency to hold on to first impressions, a response bias that can lead to inaccurate conclusions. Participants are encouraged to keep an open mind, postpone firm judgments until adequate evidence is available, and remain receptive to alternative explanations. |
| Module 4  To empathize (I) | ToM (1^st^ order)  Emotion perception | To emphasize that, while facial expressions are indicators of a person’s emotions and mental state, alone they can be misleading and should be interpreted alongside contextual information and individual’s personal history. Participants learn to integrate multiple contextual cues to improve social reasoning and to better understand others’ emotions and intentions. |
| Module 5  Memory | Overconfidence in errors | To learn that memory is constructive rather than a precise recording of past experiences. Through examples of common false memories and tasks that reliably elicit memory errors, participants practice how easily vivid-seeming recollections can be distorted. Participants are encouraged to question memories and to seek additional evidence, especially in important interpersonal situations. |
| Module 6  To empathize (II) | ToM (2^nd^ order)  Need for closure | To learn that many social situations do not allow definitive conclusions. This ambiguity can feel uncomfortable, especially for those with a strong need for closure, but it highlights the fallibility of social decision making and the importance of identifying additional information to justify any given interpretation. |
| Module 7  Jumping to conclusions (II) | JTC  Liberal acceptance | To emphasizes the disadvantage of hasty decision-making and the need to invest adequate time in solving complex problems. Participants view paintings or illustrations and must select the correct title from four options. While some answers become evident quickly, others require careful, detailed inspection. The exercises show how superficial reasoning can lead to errors, whereas slower, more deliberate evaluation helps reveal decisive features that might otherwise go unnoticed. |
| Module 8  Mood and self-esteem | Negative cognitive beliefs and emotions | This module introduces cognitive biases that contribute to depression and low self-esteem. After reviewing common symptoms, causes, and treatment options, participants explore typical depressive thinking patterns, such as overgeneralization and selective abstraction, and practice generating more balanced interpretations. The module emphasizes that these cognitive biases are learnable and changeable, and it provides strategies for reshaping negative self-beliefs and improving mood. |

*Abbreviations:* BADE, Bias against disconfirmatory evidence; JTC, Jumping to Conclusions; ToM, Theory of Mind;.

**Online Resource 5: Effects of sex and intervention group on neurocognitive variables over time, unadjusted**

|  | **Psychoeducational group** | | | | **MCT group** | | | | **Comparison between groups**  **(baseline *vs* follow-up)** | | | | |
| --- | --- | --- | --- | --- | --- | --- | --- | --- | --- | --- | --- | --- | --- |
|  | Baseline  *Mean (SD)* | | Follow-up  *Mean (SD)* | | Baseline  *Mean (SD)* | | Follow-up  *Mean (SD)* | | *P-value (effect size^a^)* | | | | |
|  | Men | Women | Men | Women | Men | Women | Men | Women | Group effect | | Sex effect | Group*Sex | |
| **CPT^c^** |  | | | | | | | | | | | | |
| Omissions | 94.14  (122.19) | 57.14  (16.02) | 81.04  (70.85) | 68.66  (42.23) | 90.10  (54.23) | 121.66  (145.84) | 62.38  (24.74) | 150.36  (315.08) | .952 (.000) | | .062 (.049) | | .460 (.008) |
| Commissions | 52.84  (12.47) | 57.02  (11.25) | 52.07  (11.70) | 59.77  (10.26) | 55.05  (12.49) | 55.81  (11.58) | 55.39  (13.29) | 51.42  (14.97) | .276 (.017) | | .827 (.001) | | .139 (.031) |
| Hit Index Reaction Time | 58.02  (13.80) | 52.54  (9.86) | 60.64  (17.34) | 55.81  (13.91) | 57.42  (16.37) | 59.62  (21.64) | 51.26  (12.71) | 57.25  (22.64) | ***.044* (.057)*** | | .529 (.006) | | .655 (.003) |
| **TMT^c^** |  | | | | | | | | | | | | |
| TMT-A | 71.87  (19.66) | 59.10  (11.46) | 61.89  (19.09) | 53.59  (12.87) | 63.63  (21.71) | 62.70  (14.17) | 58.86  (13.68) | 57.48  (14.44) | .398 (.010) | | .537 (.005) | | .450 (.008) |
| TMT-B | 79.70  (27.99) | 58.27  (11.17) | 71.08  (30.01) | 56.89  (20.77) | 77.91  (31.06) | 64.71  (15.90) | 66.10  (29.30) | 61.90  (21.35) | .639 (.003) | | .101 (.037) | | .857 (.000) |
| **WCST^c^** |  | | | | | | | | | | | | |
| Perseverative errors | 46.00  (13.71) | 42.80  (8.39) | 48.93  (8.43) | 46.40  (6.95) | 43.35  (7.19) | 45.73  (8.62) | 49.30  (8.99) | 45.73  (9.86) | .920 (.000) | .350 (.013) | | | .242 (.020) |
| Non-perseverative errors | 45.07  (13.81) | 42.80  (8.70) | 45.63  (11.25) | 44.90  (8.58) | 44.46  (12.56) | 40.65  (8.43) | 46.16  (10.51) | 48.04  (9.76) | .369 (.012) | .384 (.011) | | | .293 (.016) |
| Total errors | 44.48  (14.30) | 41.56  (7.54) | 46.56  (10.78) | 45.22  (6.98) | 41.04  (8.61) | 45.82  (6.37) | 48.61  (11.41) | 46.73  (13.84) | .647 (.003) | .397 (.011) | | | .170 (.028) |
| **Stroop Test^c^** |  | | | | | | | | | | | | |
| Word | 41.15  (9.56) | 44.67  (7.27) | 40.81  (10.41) | 44.33  (10.39) | 41.36  (11.64) | 45.40  (10.23) | 44.21  (11.17) | 51.60  (14.69) | ***.029* (.064)*** | .446 (.008) | | | .446 (.008) |
| Color | 34.37  (9.16) | 36.67  (8.21) | 36.44  (9.55) | 37.92  (8.67) | 39.75  (9.66) | 40.00  (7.54) | 39.39  (9.05) | 45.10  (15.89) | .725 (.002) | .253 (.018) | | | .123 (.032) |
| Word-Color | 45.74  (13.82) | 43.42  (10.31) | 41.81  (10.07) | 44.67  (11.79) | 48.00  (14.13) | 49.30  (15.87) | 44.93  (11.22) | 43.60  (9.07) | .341 (.012) | .690 (.002) | | | .223 (.020) |
| Interference | 54.67  (10.57) | 53.83  (6.94) | 53.89  (8.47) | 54.92  (10.02) | 56.89  (11.53) | 56.80  (15.01) | 52.71  (7.58) | 48.80  (8.74) | ***.031* (.062)*** | .731 (.002) | | | .320 (.014) |
| **WAIS-III Digit Span Subtest^c^** | 44.30  (11.99) | 43.69  (9.23) | 46.84  (12.42) | 45.75  (11.55) | 42.00  (8.81) | 48.17  (9.28) | 41.31  (8.48) | 47.42  (12.52) | .130 (.031) | .891 (.000) | | | .914 (.000) |
| **CVLT^c^** |  | | | | | | | | | | | | |
| Immediate recall |  | | | | | | | | | | | | |
| *First presentation* | 37.94  (9.69) | 47.38  (13.84) | 41.34  (8.98) | 45.62  (17.07) | 38.17  (9.95) | 41.05  (8.21) | 44.82  (10.32) | 46.28  (8.81) | ***.036* (.057)*** | .173 (.025) | | | .437 (.008) |
| *Fifth presentation* | 33.62  (17.04) | 43.50  (17.15) | 37.38  (18.27) | 46.10  (13.04) | 32.70  (15.36) | 40.35  (14.50) | 37.89  (16.69) | 40.40  (14.91) | .838 (.001) | .253 (.017) | | | .470 (.007) |
| *Total (A1-A5)^b^* | 32.43 (13.36) | 45.14 (13.24) | 38.17 (14.59) | 44.32 (14.16) | 33.45 (13.06) | 38.10 (8.83) | 38.42 (14.07) | 41.98 (14.95) | .407 (.009) | .108 (.034) | | | .249 (.018) |
| Free recall |  | | | | | | | | | | | | |
| *Short-term* | 34.80 (13.79) | 46.15 (11.98) | 40.47 (14.35) | 45.87 (13.96) | 34.90 (16.14) | 38.90 (11.00) | 37.03 (14.95) | 43.56 (15.65) | .757 (.001) | .447 (.008) | | | .063 (.045) |
| *Long-term* | 33.01 (15.22) | 42.39 (12.37) | 37.66 (19.01) | 44.79 (15.40) | 31.30 (16.55) | 39.12 (11.64) | 36.08 (17.52) | 42.28 (16.52) | .843 (.001) | .398 (.010) | | | .890 (.000) |
| Cued recall |  | | | | | | | | | | | | |
| *Short-term* | 34.05 (13.97) | 41.24 (14.30) | 40.92 (16.63) | 41.62 (17.60) | 32.63 (14.81) | 38.35 (13.35) | 36.67 (15.16) | 37.75 (18.48) | .450 (.008) | ***.030* (.061)*** | | | .714 (.002) |
| *Long-term* | 32.40 (17.83) | 40.32 (16.30) | 39.83 (17.87) | 43.52 (17.66) | 30.26 (16.82) | 35.31 (12.90) | 35.95 (16.75) | 37.81 (17.58) | .602 (.004) | .118 (.032) | | | .826 (.001) |
| Semantic clustering |  | | | | | | | | | | | | |
| \| *Immediate recall* \|  \|  \|  \|  \|  \|  \| \| --- \| --- \| --- \| --- \| --- \| --- \| --- \| | 39.79 (6.85) | 48.45 (10.35) | 47.53 (28.87) | 46.47 (10.36) | 41.53 (6.09) | 40.98 (5.77) | 42.58 (5.90) | 45.32 (12.82) | .968 (.000) | .479 (.007) | | | .155 (.027) |
| \| *Short-term free recall* \|  \|  \|  \|  \|  \|  \| \| --- \| --- \| --- \| --- \| --- \| --- \| --- \| | 39.52 (7.62) | 48.74 (8.91) | 42.65 (9.87) | 46.40 (11.13) | 41.56 (7.93) | 43.76 (7.71) | 43.25 (9.10) | 43.50 (9.48) | .887 (.000) | .091 (.038) | | | .421 (.009) |
| \| *Long-term free recall* \|  \|  \|  \|  \|  \|  \| \| --- \| --- \| --- \| --- \| --- \| --- \| --- \| | 38.33 (8.09) | 45.12 (8.39) | 43.61 (12.24) | 46.61 (10.21) | 40.25 (8.44) | 42.48 (7.95) | 41.01 (8.76) | 44.51 (10.94) | .375 (.010) | .572 (.004) | | | .259 (.017) |
| \| Serial clustering \|  \|  \|  \|  \|  \|  \| \| --- \| --- \| --- \| --- \| --- \| --- \| --- \| |  | | | | | | | | | | | | |
| \| *Immediate recall* \|  \|  \|  \|  \|  \|  \| \| --- \| --- \| --- \| --- \| --- \| --- \| --- \| | 49.18 (7.22) | 50.59 (6.70) | 49.10 (10.05) | 49.58 (4.41) | 52.22 (16.80) | 53.78 (6.52) | 50.90 (9.21) | 52.42 (14.31) | .803 (.001) | .881 (.000) | | | .892 (.000) |
| \| *Short-term free recall* \|  \|  \|  \|  \|  \|  \| \| --- \| --- \| --- \| --- \| --- \| --- \| --- \| | 50.68 (6.22) | 46.88 (3.72) | 49.15 (6.47) | 51.28 (11.48) | 53.55 (14.07) | 47.03 (3.86) | 50.01 (7.26) | 53.39 (12.12) | .992 (.000) | ***.005* (.103)*** | | | .467 (.007) |
| \| *Long-term free recall* \|  \|  \|  \|  \|  \|  \| \| --- \| --- \| --- \| --- \| --- \| --- \| --- \| | 47.50 (5.45) | 52.27 (6.13) | 48.75 (5.98) | 52.18 (8.14) | 50.08 (12.46) | 51.33 (8.92) | 53.08 (10.99) | 53.19 (15.10) | .466 (.007) | .622 (.003) | | | .969 (.000) |
| \| Perseverations \|  \|  \|  \|  \|  \|  \| \| --- \| --- \| --- \| --- \| --- \| --- \| --- \| | 47.68 (10.37) | 44.45 (6.90) | 47.87 (10.73) | 44.74 (6.75) | 54.57 (16.78) | 52.59 (9.58) | 48.34 (8.85) | 51.37 (12.58) | .135 (.030) | .334 (.012) | | | .355 (.011) |
| \| Intrusions \|  \|  \|  \|  \|  \|  \| \| --- \| --- \| --- \| --- \| --- \| --- \| --- \| |  | | | | | | | | | | | | |
| \| *Free recall* \|  \|  \|  \|  \|  \|  \| \| --- \| --- \| --- \| --- \| --- \| --- \| --- \| | 50.28 (10.03) | 46.51 (6.32) | 47.90 (8.54) | 51.71 (11.83) | 53.25 (13.39) | 47.68 (7.55) | 49.50 (9.17) | 47.69 (5.56) | .190 (.023) | ***.025* (.065)*** | | | .445 (.008) |
| \| *Cued recall* \|  \|  \|  \|  \|  \|  \| \| --- \| --- \| --- \| --- \| --- \| --- \| --- \| | 52.33 (10.15) | 52.44 (14.08) | 49.19 (10.30) | 49.88 (7.87) | 53.44 (12.08) | 51.12 (14.36) | 52.16 (11.97) | 48.27 (6.52) | .789 (.001) | .865 (.000) | | | .715 (.002) |
| Recognition |  | | | | | | | | | | | | |
| \| *Accuracy* \|  \|  \|  \|  \|  \|  \| \| --- \| --- \| --- \| --- \| --- \| --- \| --- \| | 34.61 (26.19) | 45.50 (17.95) | 36.44 (28.81) | 45.82 (12.57) | 35.88 (22.27) | 43.43 (15.18) | 40.34 (17.35) | 46.62 (17.25) | .529 (.005) | .748 (.001) | | | .979 (.000) |
| \| *False positives* \|  \|  \|  \|  \|  \|  \| \| --- \| --- \| --- \| --- \| --- \| --- \| --- \| | 59.97 (17.81) | 49.68 (8.94) | 57.87 (25.08) | 49.66 (6.74) | 57.40 (18.03) | 50.22 (6.99) | 54.28 (12.14) | 51.15 (14.40) | .992 (.000) | .435 (.008) | | | .804 (.001) |

*Abbreviations:* CPT, Continuous Performance Test; CVLT, California Verbal Learning Test; MCT, Metacognitive Training; SD, Standard Deviation; TMT, Trail Making test; WAIS-III, Wechsler Adult Intelligence Scale; WCST, Wisconsin Card Sorting Test.

*^a^*Effect sizes are provided with partial eta square (η_p_^2^)

*^b^*Total = sum of trials A1 to A5

*^c^*Values are shown as T scores (mean = 50, SD = 10)

*Level of significance <0.05
